# Sign-Entropy Regularization for Personalized Federated Learning

**DOI:** 10.3390/e27060601

**Published:** 2025-06-04

**Authors:** Koffka Khan

**Affiliations:** Department of Computing and Information Technology, The University of the West Indies, St. Augustine 350462, Trinidad and Tobago; koffka.khan@sta.uwi.edu; Tel.: +1-868-662-2002

**Keywords:** federated learning, entropy regularization, gradient sign patterns, personalization, polynomial root geometry

## Abstract

Personalized Federated Learning (PFL) seeks to train client-specific models across distributed data silos with heterogeneous distributions. We introduce *Sign-Entropy Regularization* (SER), a novel entropy-based regularization technique that penalizes excessive directional variability in client-local optimization. Motivated by Descartes’ Rule of Signs, we hypothesize that frequent sign changes in gradient trajectories reflect complexity in the local loss landscape. By minimizing the entropy of gradient sign patterns during local updates, SER encourages smoother optimization paths, improves convergence stability, and enhances personalization. We formally define a differentiable sign-entropy objective over the gradient sign distribution and integrate it into standard federated optimization frameworks, including FedAvg and FedProx. The regularizer is computed efficiently and applied post hoc per local round. Extensive experiments on three benchmark datasets (FEMNIST, Shakespeare, and CIFAR-10) show that SER improves both average and worst-case client accuracy, reduces variance across clients, accelerates convergence, and smooths the local loss surface as measured by Hessian trace and spectral norm. We also present a sensitivity analysis of the regularization strength ρ and discuss the potential for client-adaptive variants. Comparative evaluations against state-of-the-art methods (e.g., Ditto, pFedMe, momentum-based variants, Entropy-SGD) highlight that SER introduces an orthogonal and scalable mechanism for personalization. Theoretically, we frame SER as an information-theoretic and geometric regularizer that stabilizes learning dynamics without requiring dual-model structures or communication modifications. This work opens avenues for trajectory-based regularization and hybrid entropy-guided optimization in federated and resource-constrained learning settings.

## 1. Introduction

Federated Learning (FL) enables training a shared model on distributed data held by clients without centralizing the data [1]. The standard Federated Averaging (FedAvg) algorithm [1] iteratively aggregates local model updates and has shown remarkable robustness even under non-independent and identically distributed (non-iid) data and across clients [1]. However, with highly heterogeneous data, a single global model can underperform for individual clients. This has spurred Personalized Federated Learning (PFL), which learns client-specific models to better fit local data while still leveraging global knowledge. Key challenges in PFL include handling data heterogeneity, convergence stability, and generalization to new clients [2].

Recent work has emphasized the importance of robust generalization in data-scarce or heterogeneous environments. For instance, metric learning techniques have been proposed to overcome representation collapse under few-shot constraints [3]. Similarly, in federated settings, personalization methods aim to tailor models to each client’s local data while maintaining some shared structure [4,5,6]. Our proposed Sign-Entropy Regularization (SER) contributes to this growing area by improving optimization stability and generalization under non-iid and low-data regimes via directional entropy control rather than through explicit meta-learning or model decoupling.

Meanwhile, regularization techniques are crucial in machine learning to prevent overfitting and improve generalization. Entropy-based regularization is a class of methods incorporating information-theoretic measures (entropy) into loss functions in order to encourage desirable properties such as uncertainty or flat minima. For example, adding an entropy term to policy gradients in reinforcement learning encourages exploration [7], while penalizing low-entropy (overconfident) predictions in supervised learning acts as a regularizer that improves generalization [8]. In optimization, methods such as Entropy-SGD are intended to bias training towards wide and flat minima by adding a local entropy term to the objective [9]. These approaches inspire us to consider entropy-based penalties on aspects of the federated training dynamics. In addition to average performance, our approach also improves fairness across clients, reducing performance disparity and improving worst-case accuracy. We evaluate this effect using standard deviation and 10th percentile metrics, and further validate our method’s stability through a sensitivity analysis of the regularization strength ρ. To capture the optimization geometry, we introduce curvature-based diagnostics (Hessian trace and top eigenvalue), which confirm that SER leads to flatter loss landscapes and more stable descent directions.

In this work, we propose a novel Sign-Entropy Regularization (SER) technique for personalized federated learning. Our approach is motivated by a geometric analogy to polynomial root-finding: Descartes’ Rule of Signs in polynomial theory states that the number of positive real roots is bounded by the number of sign changes in the polynomial’s coefficients [10]. By analogy, frequent sign changes in the gradient signal of a loss function may indicate numerous local minima or an irregular loss landscape. We hypothesize that constraining the “sign entropy” of client gradients—a measure of the variability or unpredictability in the sign patterns of gradients—can lead to smoother optimization trajectories and better personalized models. In essence, we treat the pattern of gradient sign changes during local training as an analog to a polynomial’s sign pattern, then introduce a regularizer that penalizes excessive sign changes (high entropy in the sign sequence). This polynomial root geometry perspective provides a novel lens through which to examine federated optimization; just as fewer sign changes in a polynomial imply fewer possible roots, a more regularized sign pattern in gradients may imply fewer spurious optima and more stable convergence. We revisit this analogy more formally in the Discussion section, clarifying its relevance under convexity assumptions and one-dimensional trajectories.

The contributions of this paper are threefold. First, we review foundational literature on entropy-based regularization, personalized federated learning algorithms, polynomial sign change theory, and federated optimization theory while drawing connections between these areas. Second, we formulate the sign-entropy regularization term derived from sign-change behavior in client-local training. We describe how this term can be integrated into the local objective of each client to guide training toward flatter minima and align with global updates. A theoretical discussion is provided linking our approach to Descartes’ rule [10] and outlining how sign-entropy might relate to generalization bounds in federated settings (e.g., by controlling the hypothesis space complexity via sign-change constraints). Third, we design extensive experiments on standard federated benchmarks—FEMNIST (image classification), Shakespeare (next-character prediction), and CIFAR-10 (image classification)—under heterogeneous data distributions. We compare our SER-augmented FedAvg (and FedProx) against baseline methods including FedAvg [1], FedProx [11], pFedMe [12], and Ditto [4]. Results show that SER consistently improves personalized accuracy on each client and accelerates convergence in many cases. To offer insight into why sign-entropy regularization is effective, we provide visualizations of the training process, including the evolution of each client’s gradient sign entropy, accuracy curves over training rounds, and global model aggregation behavior.

## 2. Related Work

### 2.1. Entropy-Based Regularization in Learning and Optimization

Regularization via entropy has been explored in various domains of machine learning. In reinforcement learning, adding an entropy bonus to the reward objective (i.e., maximizing policy entropy) is a common technique to maintain exploration and prevent premature convergence to deterministic policies [7]. For instance, the A3C and PPO algorithms include an entropy term to encourage the policy’s action distribution to stay broad (high entropy), thereby avoiding suboptimal deterministic choices. In supervised learning, confidence penalty methods add a term to penalize low-entropy model output distributions [8]. Pereyra et al. [8] introduced a regularizer that adds the negative of the prediction entropy to the loss, thereby discouraging overconfident (near 0 or 1 probability) predictions. This was shown to improve generalization across image recognition, language modeling, and speech tasks by preventing the network from becoming too sure about incorrect predictions.

In the context of optimization landscapes, entropy-based regularization has been used to find flatter minima. Entropy-SGD by Chaudhari et al. [9] is a notable example that augments the loss with a local entropy term, which effectively smooths the loss surface. The algorithm employs stochastic Langevin dynamics internally, biasing gradient descent towards wide valleys in the loss landscape [9]. By favoring parameters that lie in regions of low curvature (high local entropy), Entropy-SGD improves generalization and demonstrates more stable convergence than standard SGD [9]. This idea of using entropy to favor flat minima is conceptually related to our approach, in which we seek to regularize the signs of gradients to avoid the oscillations associated with narrow or sharp minima.

A prominent entropy-based optimization method that deserves particular attention is Entropy-SGD  [9]. Entropy-SGD augments the loss objective by biasing optimization towards wide and flat regions of the landscape. This is achieved by approximating a local entropy around the current iteration using stochastic Langevin dynamics, effectively replacing the standard objective L(w) with a smoothed version:

$$L_{\mathrm{ESGD}}\left(w\right)=-\log\int \exp\left(-L(w+\xi )/\tau \right)d\xi$$

where τ is a temperature hyperparameter and ξ is Gaussian noise. This formulation encourages convergence to flatter minima by penalizing sharp curvature in the loss surface.

Our proposed Sign-Entropy Regularization (SER) differs in both its goal and mechanism. Rather than modifying the loss function directly, SER penalizes the entropy of *gradient sign patterns* observed during client-local training. In this way, it regularizes the optimization *trajectory*, promoting stability and consistency in the update direction over time. While Entropy-SGD focuses on the *geometry* of the loss landscape, SER targets the *dynamics* of descent, making the two approaches orthogonal and potentially complementary.

It is important to note that entropy regularization can be applied to different quantities, for instance, model predictions, weight distributions, or in our case, gradient signals. Our work appears to be the first to apply an entropy-based measure to gradient sign patterns as a form of regularization. By doing so, we connect to the intuition that a model which oscillates less (in terms of gradient sign) might reside in a flatter part of the loss surface. This connection between sign stability and flat minima underpins the proposed sign-entropy regularizer.

### 2.2. Gradient-Based Regularization Techniques

Two widely used regularization strategies in optimization are Stochastic Gradient Descent (SGD) with momentum and gradient norm regularization. While both are conceptually related to our proposed SER in that they influence the dynamics of parameter updates, they operate on different aspects of the gradient behavior.

SGD with Momentum was first introduced in the foundational work by Rumelhart, Hinton, and Williams [13], where the update vector accumulates a weighted average of past gradients:

$$v^{\left(k\right)}=\gamma v^{\left(k-1\right)}+\eta \cdot \nabla \ell \left(w^{\left(k\right)}\right),\;w^{\left(k+1\right)}=w^{\left(k\right)}-v^{\left(k\right)}.$$

This formulation implicitly regularizes the velocity of updates, discouraging abrupt directional changes and smoothing the optimization path. Momentum shares the high-level goal of our method, that is, promoting consistency in the update trajectory, but does so by penalizing *magnitude-based changes* in the parameter space.

Gradient Norm Regularization, in contrast, penalizes large gradients directly by augmenting the loss with terms such as $\lambda \cdot {∥\nabla \ell \left(w\right)∥}^{2}$ [14]. This approach constrains the scale of updates, helping to prevent instability or overshooting during optimization. Although our method also introduces a gradient-dependent penalty, it operates on the *distribution of gradient signs* rather than on their norm. Thus, SER is scale-invariant and focuses on directional entropy, a qualitatively different regularization objective.

In overview, while both momentum and gradient norm regularization aim to stabilize training, our proposed SER introduces a novel geometric and information-theoretic perspective by minimizing the entropy of sign trajectories. This complements existing approaches and may be combined with them in future hybrid strategies.

### 2.3. Personalized Federated Learning Methods

Federated Averaging (FedAvg) [1] remains the cornerstone of FL algorithms. FedAvg simply averages client updates, and works surprisingly well even on non-iid data; however, it learns a single global model that may not generalize to each client’s distribution. To tackle this limitation, numerous personalized FL approaches have been proposed. FedProx [11] introduces a proximal term in each client’s loss in order to restrict how far local model updates can diverge from the global model. This stabilizes convergence under heterogeneity by preventing extreme local updates; indeed FedProx has shown more robust behavior than FedAvg on highly heterogeneous data [11]. In our experiments, we use FedProx as a baseline, as it represents a simple but strong personalization approach via regularizing the update step.

Another line of work uses model interpolation and meta-learning. pFedMe (Dinh et al., 2020) uses Moreau envelopes to decouple personalized model optimization from global model updates [12]. Each client solves a regularized local problem in which the solution is an intermediate personalized model, then the global model is updated towards these personalized models. pFedMe achieves state-of-the-art convergence rates for both strongly convex and certain nonconvex objectives [12]. We compare our approach to pFedMe in order to represent the class of algorithms in which each client maintains its own model close to the global model. Similarly, Ditto (Li et al., 2021) trains a global model for fairness while simultaneously learning personalized models via a simple regularization; each client’s objective is augmented with a term $|w_{i}-w_{\mathrm{global}}{|}^{2}$ to keep it close to the global parameters [4]. Ditto demonstrates strong performance on fairness (uniformity of accuracy across clients) and robustness against poisoning attacks [4]. In essence, Ditto’s approach is to split the difference between a global and local model, which is a paradigm that our method also implicitly follows. By regularizing gradient signs, our client models do not stray too wildly, which is somewhat akin to staying closer to a shared optimum but without an explicit parameter norm penalty.

Other notable PFL methods include FedAMP (Huang et al., 2021), which uses attentive message passing between similar clients to personalize models [15]. FedAMP clusters the learning process; clients with analogous data can collaborate more by effectively weighting each other’s updates more strongly. This yields improved performance on non-iid data, though at the cost of extra communication to estimate client similarity [15]. Another approach is specialization in parts of the model; e.g., FedPer and FedRep share some layers (such as a representation layer) while others are personalized, which can be effective when feature distributions vary but high-level representations can be shared. Furthermore, FedBN (Li et al., 2021) addresses feature distribution heterogeneity by keeping the batch normalization layers local to each client while aggregating the rest of the network [16]. FedBN has achieved notable gains in computer vision FL tasks with feature shift by not forcing a single normalization statistic across clients [16].

Our proposed method is complementary to many of these approaches. Most personalized FL algorithms introduce additive regularization terms or alternate updates to achieve personalization (proximal terms in FedProx [11] and Ditto [4], meta-learning in pFedMe [12], etc.). We introduce a different kind of regularization based on gradient sign patterns, which could theoretically be combined with these methods. In this paper, however, we treat our method as a modification of the FedAvg [1]/FedProx [11] style of training (i.e., an additional term in the local loss function) and compare it against the above baseline methods to evaluate its effectiveness.

### 2.4. Polynomial Root Geometry and Sign Patterns

Our work is inspired by an analogy between the behavior of gradients during training and the sign patterns of polynomial coefficients. Descartes’ Rule of Signs is a classical result that provides an upper bound on the number of positive real roots of a polynomial based on the number of sign changes in its sequence of coefficients. In simple terms, if a polynomial’s coefficients (ordered by descending power) change their sign *V* times, then the polynomial has at most *V* positive roots [10]. For example, the polynomial $f\left(x\right)=+2x^{3}-4x^{2}-5x+3$ has coefficients (+2,−4,−5,+3) which have three sign changes, meaning that there can be at most three positive real roots (the exact number of positive roots will be three or less, differing from three by an even number). Descartes’ rule, including various generalizations and refinements (e.g., Laguerre’s extensions), has been well-studied in mathematics [17]. It provides a link between sign patterns and solution counts, which is a source of inspiration for us; we theorize that the complexity of the optimization landscape (number of local optima) could be related to the pattern of sign changes in the gradient.

In machine learning, while polynomial root-finding is not directly at play, the concept of sign patterns has appeared in different forms. For instance, recent work by Lewandowski et al. [18] examined the sign entropy of neural network activation units over data distributions. They defined the “unit sign entropy” as a measure of how frequently a neuron’s activation is positive or negative across inputs. Linear networks have high sign entropy (about half positive and half negative outputs), whereas deep ReLU networks often have low sign entropy as neurons saturate in one regime [18]. This idea of sign entropy in activations is indirectly related to the richness of a network’s feature representations. In our case, we consider sign entropy in gradients over time, which reflects the richness or oscillations in the optimization trajectory.

Another related concept involves sign-based gradient methods (e.g., SignSGD), which compress gradients using their sign. SignSGD has been shown to work under certain conditions, but fails in heterogeneous data settings without modifications [19]. Interestingly, the failure of naive sign-based updates when using non-iid data suggests that while sign information alone is rich, it must be handled carefully. This further motivates our study of sign patterns, although we use them in a regularization term rather than for communication compression.

Sign-based gradient compression methods such as SignSGD [19] offer a contrasting but related approach to handling gradients in distributed settings. SignSGD replaces full-precision gradients with their signs in order to achieve communication efficiency, transmitting just a single bit per coordinate and performing aggregation via majority vote or sign averaging. While highly bandwidth-efficient, SignSGD has limitations under non-iid client distributions, where client gradient directions can diverge substantially.

In contrast, our SER operates as a local training-time regularizer, not a communication protocol; rather than transmitting signs, SER penalizes the entropy of sign trajectories observed across local SGD steps, thereby reducing directional oscillations and promoting smoother convergence. SER does not aim to compress gradients but rather to stabilize client-side learning. These two perspectives are complementary; SignSGD focuses on aggregation efficiency, while SER targets intra-client optimization dynamics.

We note that a hybrid method combining SER with sign-based communication (e.g., applying SER during local training and then compressing updates with SignSGD) may further improve performance in bandwidth-constrained federated systems. While such integration is outside the scope of this study, we outline it as a promising future direction in the Discussion section.

In overview, the key insight we take from polynomial geometry is this: sign changes are indicative of complexity. A function (polynomial or loss landscape) with many alternating signs might have many extrema or roots. By regulating sign changes, it might be possible to control the number of extrema. Of course, neural network loss surfaces are not polynomials in a single variable, being high-dimensional and non-convex; nonetheless, our use of sign-entropy as a measure attempts to capture a similar notion of complexity in a client’s loss landscape. To the best of our knowledge, this connection between Descartes’ rule and training dynamics is novel in federated learning. We leverage it as an analogy to justify why one might add a term that explicitly depends on the sign of gradients or updates.

### 2.5. Federated Optimization Theory and Generalization on Non-IID Data

Theoretical understanding of federated optimization has been advancing, particularly regarding convergence on non-iid data and generalization to new clients. Classical convergence analyses (e.g., for FedAvg) often assume certain smoothness and bounded variance conditions, and sometimes assume iid data to guarantee convergence. In practice, violating the iid assumption (as is typical in FL) can slow convergence or lead to biased models [20]. FedProx’s introduction was accompanied by a convergence guarantee under relaxed assumptions of data heterogeneity [11]. In addition to convergence rates, researchers have asked how well the learned model generalizes to data from unseen clients or the underlying global distribution.

Mohri et al. [21] proposed an agnostic federated learning framework which considers a worst-case mixture of client distributions in the objective. Their formulation and bounds ensure that the global model performs uniformly well across different target distributions (a form of distributional robustness). This line of work implies that a model overly fitted to the observed clients may not generalize well to new ones, highlighting the need for some regularization or robustness in the FL objective.

More recently, there have been attempts to derive generalization error bounds for federated learning that account for client heterogeneity. Hu et al. [2] provided bounds on the generalization error for both participating and held-out (unparticipating) clients using a two-level distribution framework. They showed that the generalization error can be decomposed into terms that include the divergence between client distributions. Similarly, Wei et al. [20] presented an excess risk bound for federated learning under non-iid data by decomposing the gap between the federated model and the optimal centralized model into three components: an agnostic error (due to distribution shift), a federated optimization error, and an approximation error. Their analysis identified Rademacher complexity and distribution discrepancy as key factors affecting generalization. Notably, they proposed adding regularization and a small amount of data sharing (a common sample across clients) to mitigate the discrepancy, which led to improved performance.

These theoretical works collectively suggest that controlling the complexity of local models and how much they overfit local data is crucial for both convergence and generalization in FL. Our sign-entropy regularizer is conceptually aligned with this goal; by penalizing erratic sign changes (which often accompany overfitting to noisy or idiosyncratic local patterns), we aim to restrict the local models to a smoother class of functions that still fit the data well but generalize better. While a full generalization bound for our method is beyond the scope of this paper, we hypothesize that it effectively reduces the hypothesis space complexity, perhaps in an analogous way to reducing some notion of “local Rademacher complexity” by smoothing the loss landscape [2,20]. In Section 6.1, we further discuss the potential theoretical implications of SER in federated learning as well as how it might complement existing convergence analyses.

## 3. Methodology

### 3.1. Sign-Entropy Regularization Formulation

We now introduce the proposed Sign-Entropy Regularization (SER) for personalized federated learning. Our approach modifies the local objective at each client by adding a term that captures the entropy of the sign pattern of the gradients (or updates) observed during local training. Intuitively, this term will be low when the gradient maintains a consistent sign (low variability) and high when the gradient sign frequently flips (high variability). By adding this term and minimizing the overall objective, we penalize high sign entropy, thereby discouraging frequent sign flips.

Consider a federated setting with *N* clients. Each client *i* has a local dataset $D_{i}$ and aims to learn a model parameter vector $w_{i}\in R^{d}$. In standard FedAvg/FedProx, during each round the client solves (approximately) the problem

$$\min_{w}F_{i}\left(w\right)=\ell_{i}\left(w\right)+\frac{\mu }{2}{∥w-w_{g}∥}^{2},$$

where $\ell_{i}\left(w\right)$ is the local data loss and $w_{g}$ is the current global model (note that μ=0 for FedAvg). We modify this objective to

$$\min_{w}F_{i}^{\mathrm{SER}}\left(w\right)=\ell_{i}\left(w\right)+\rho \cdot \Omega_{i}\left(w\right),$$

where $\Omega_{i}\left(w\right)$ is the sign-entropy regularizer for client *i* and ρ>0 is a hyperparameter controlling its strength. We now define $\Omega_{i}\left(w\right)$ precisely.

During the local update on client *i*, suppose that we perform *K* steps of Stochastic Gradient Descent (SGD) on $\ell_{i}$. Let $g_{i}^{\left(k\right)}=\nabla \ell_{i}\left(w_{i}^{\left(k\right)}\right)$ be the gradient at step *k* (where k=1,…,K and $w_{i}^{\left(1\right)}$ is the initial model from the server, $w_{i}^{\left(K+1\right)}$ the final model after local training). We focus on the sign of each component of the gradient. We denote by $\mathrm{sgn}\left(g_{i,j}^{\left(k\right)}\right)\in \left\{+1,-1\right\}$ the sign of the *j*-th component of client *i*’s gradient at step *k*.

Over the *K* local iterations, client *i* will observe a sequence of signs for each parameter *j*:

$$(\mathrm{sgn}\left(g_{i,j}^{\left(1\right)}\right),\mathrm{sgn}\left(g_{i,j}^{\left(2\right)}\right),\ldots ,\mathrm{sgn}\left(g_{i,j}^{\left(K\right)}\right)).$$

From this sequence, we derive a measure of entropy. There are a few choices for defining entropy on a sequence of binary values:

- **Sign Distribution Entropy:** We can ignore the order of flips and simply compute the proportion of time the sign was positive versus negative. Let
  $$p_{i,j}=\frac{1}{K}\sum_{k=1}^{K}1\left\{\mathrm{sgn}\left(g_{i,j}^{\left(k\right)}\right)=+1\right\}$$
  be the fraction of iterations where the *j*-th gradient was positive (and let $1-p_{i,j}$ be the fraction it was negative). The entropy of the sign distribution for parameter *j* is defined as follows:
  $$H_{i,j}=-\left[p_{i,j}\log p_{i,j}+\left(1-p_{i,j}\right)\log\left(1-p_{i,j}\right)\right].$$
  This quantity is 0 if the sign was always the same (+1 or −1) over local training and is maximal (1 bit of entropy) if the sign was positive half the time and negative half the time (completely unpredictable sign).
- **Sign Change Entropy:** Alternatively, we can look at the sequence of sign changes. Defining
  $$\Delta_{i,j}^{\left(k\right)}=1\left\{\mathrm{sgn}\left(g_{i,j}^{\left(k\right)}\right)\neq \mathrm{sgn}\left(g_{i,j}^{\left(k-1\right)}\right)\right\}\;\mathrm{for}\;k=2,\ldots ,K$$
  as an indicator that a flip occurred at step *k* for parameter *j*, we compute the frequency of flips as follows:
  $$q_{i,j}=\frac{1}{K-1}\sum_{k=2}^{K}\Delta_{i,j}^{\left(k\right)}$$
  and define the corresponding entropy
  $$-\left[q_{i,j}\log q_{i,j}+\left(1-q_{i,j}\right)\log\left(1-q_{i,j}\right)\right].$$
  This would be 0 if no sign changes occurred (or if a sign change happened at every step, in which case $q_{i,j}=1$ and entropy is also 0) and maximal when $q_{i,j}=0.5$ (the sign flips half the time). This measure directly focuses on oscillations.

After preliminary exploration, we opted for the simpler sign distribution entropy $H_{i,j}$ as our regularizer, since it more directly measures the balance of the gradient direction. Thus, we define

(1) $$\Omega_{i}\left(w\right)=\frac{1}{d}\sum_{j=1}^{d}H_{i,j}=-\frac{1}{d}\sum_{j=1}^{d}\left[p_{i,j}\log p_{i,j}+\left(1-p_{i,j}\right)\log\left(1-p_{i,j}\right)\right],$$

where $\Omega_{i}\left(w\right)$ is the average entropy of gradient signs across the *d* dimensions of the model for client *i* during its local update.

In implementation, we accumulate the counts of positive vs. negative gradient occurrences for each parameter over the *K* local mini-batches. To avoid undefined values, we smooth the counts (ensuring neither $p_{i,j}$ nor $1-p_{i,j}$ is zero); in practice, this was rarely an issue except in trivial cases.

### 3.2. Differentiability of the Sign-Entropy Term

As originally defined, the entropy term $\Omega_{i}\left(w\right)$ depends on $p_{i,j}$, that is, the empirical proportion of times the *j*-th gradient component is positive during local updates:

$$p_{i,j}=\frac{1}{K}\sum_{k=1}^{K}1\left\{\mathrm{sgn}(g_{i,j}^{\left(k\right)})=+1\right\}.$$

However, the indicator function 1{·} and the sign function sgn(·) are both non-differentiable, making this formulation unsuitable for gradient-based optimization.

To ensure differentiability, we introduce a smooth surrogate for the indicator. Specifically, we approximate

$$1\left\{g_{i,j}^{\left(k\right)}>0\right\}\approx \sigma \left(\beta \cdot g_{i,j}^{\left(k\right)}\right),$$

where $\sigma \left(x\right)=\frac{1}{1+e^{-x}}$ is the sigmoid function and β>0 controls the sharpness of the transition. In our implementation, we used β=5 to balance smoothness and fidelity.

Thus, we compute the differentiable surrogate:

$${\tilde{p}}_{i,j}=\frac{1}{K}\sum_{k=1}^{K}\sigma \left(\beta \cdot g_{i,j}^{\left(k\right)}\right)$$

and define the smoothed entropy:

$$H_{i,j}=-{\tilde{p}}_{i,j}\log{\tilde{p}}_{i,j}-\left(1-{\tilde{p}}_{i,j}\right)\log\left(1-{\tilde{p}}_{i,j}\right).$$

This entropy is differentiable with respect to each local gradient $g_{i,j}^{\left(k\right)}$, enabling full backpropagation of the SER loss term. We used this smoothed entropy in all experiments and found that it improves both numerical stability and optimization effectiveness compared to alternatives such as hard-counting with stop-gradient.

This implementation ensures that the regularization term $\Omega_{i}\left(w\right)$ is tractable and differentiable, facilitating seamless integration into client-local training via gradient-based methods.

#### 3.2.1. Gradient Computation and Approximation of $\nabla \Omega_{i}$

To enable gradient-based optimization, we approximate the gradient of the sign-entropy regularizer $\Omega_{i}$ using a smooth surrogate for the sign indicator. As described earlier, we compute a differentiable proxy for the sign frequency:

$${\tilde{p}}_{i,j}=\frac{1}{K}\sum_{k=1}^{K}\sigma \left(\beta \cdot g_{i,j}^{\left(k\right)}\right)$$

where $\sigma \left(x\right)=\frac{1}{1+e^{-\beta x}}$ is the sigmoid function and β is a sharpness parameter (set to 5 in our experiments).

The entropy of this distribution is then

$$H_{i,j}=-{\tilde{p}}_{i,j}\log{\tilde{p}}_{i,j}-\left(1-{\tilde{p}}_{i,j}\right)\log\left(1-{\tilde{p}}_{i,j}\right),$$

and the regularizer $\Omega_{i}$ is obtained by averaging over all dimensions. This formulation is fully differentiable with respect to $g_{i,j}^{\left(k\right)}$, and hence with respect to w through the gradient chain rule.

We compute the gradient $\nabla \Omega_{i}$ by standard backpropagation through this entropy function. We do not use stop-gradient approximations or stochastic sampling methods. As a result, the approximation introduces no bias in the gradient computation, and training remains end-to-end differentiable.

We empirically observed that using this smooth approximation significantly improved stability compared to earlier experiments that used stop-gradient or hard thresholding strategies. Therefore, all reported results use this unbiased differentiable implementation.

#### 3.2.2. Why This Definition?

It captures the intuition that if a client’s gradients in a particular dimension are consistently pointing the same way (either consistently positive or consistently negative), then that dimension might be on a stable downhill slope toward a minimum (or climbing out of a maximum). If instead the gradient’s sign is frequently changing, then the loss surface in that dimension might be oscillating, indicative of either bouncing around a local extremum or noise in gradients. High sign entropy could also mean that the model parameter has not decided on a descent direction due to conflicting pulls (possibly from a non-convex landscape with multiple minima nearby).

By minimizing $\Omega_{i}$, we encourage low entropy in gradient signs, meaning that each parameter tends to have a consistent descent direction during local training. This can be seen as encouraging a locally quasi-convex behavior: if each component of the gradient does not change sign, then the local loss might be closer to convex in that region (i.e., no multiple crossings of zero slope). In polynomial terms, we discourage multiple sign changes in the “derivative” (gradient), which might limit the number of distinct minima in a client’s loss, effectively smoothing it.

It is worth noting that $\Omega_{i}$ only adds pressure, and does not explicitly force all signs to be the same across the entire training. The hyperparameter ρ balances this pressure against fitting the data. In the limit ρ→0, we recover standard FedAvg (or FedProx, if the proximal term is included separately). In the limit of very large ρ, the client would prioritize keeping gradients of one sign even if the loss reduction is small, which could lead to underfitting the local data. We expect an intermediate value of ρ to work best, and this expectation is confirmed in our experiments.

#### 3.2.3. Gradients vs. Update Trajectories

Although our method is formulated in terms of the gradient sequence $\left\{g_{i}^{\left(1\right)},g_{i}^{\left(2\right)},\ldots ,g_{i}^{\left(K\right)}\right\}$ observed during client-local training, it is important to clarify that our sign-entropy regularizer effectively operates on the *parameter update vectors*.

In typical first-order optimization methods such as Stochastic Gradient Descent (SGD), the parameter updates are provided by

$$\Delta w_{i}^{\left(k\right)}=-\eta \cdot g_{i}^{\left(k\right)}.$$

Thus, the direction of each update $\Delta w_{i}^{\left(k\right)}$ is determined by the sign of the corresponding gradient. As a result, regularizing the sign pattern of the gradients is mathematically equivalent to regularizing the sign pattern of the updates:

$$\mathrm{sgn}\left(\Delta w_{i,j}^{\left(k\right)}\right)=-\mathrm{sgn}\left(g_{i,j}^{\left(k\right)}\right).$$

From this perspective, the proposed entropy penalty on the gradient signs can be reinterpreted as a regularization mechanism that encourages *consistency in update directions* across local steps.

This aligns with established ideas in optimization such as momentum, which smooths the update trajectory by discouraging erratic directional shifts. In contrast to momentum, which penalizes the *magnitude* of directional deviation (typically in $\ell_{2}$ norm), our entropy-based regularizer penalizes the *frequency* and unpredictability of directional changes.

We make this clarification explicit in order to distinguish our approach from general gradient regularization methods as well as to emphasize its role in controlling update trajectory complexity in personalized federated settings.

### 3.3. Optimization with SER

Integrating SER into federated training involves a few modifications. Algorithm 1 summarizes the training procedure with sign-entropy regularization (assuming FedAvg-style synchronous rounds):
**Algorithm 1** FedAvg with Sign-Entropy Regularization (FedAvg-SER)**Require:** Initial global model $w_{g}^{\left(0\right)}$, learning rate η, SER weight ρ, number of clients *N* (or fraction *C* per round), number of local steps *K*
1:**for** each global round t=1,2,…,T **do**2:  Server broadcasts $w_{g}^{\left(t-1\right)}$ to selected clients3:  **for** each client *i* in selected set **do**4:   Initialize local model $w_{i}^{\left(1\right)}\leftarrow w_{g}^{\left(t-1\right)}$5:   **for** k=1 to *K* **do**6:    Compute gradient $g_{i}^{\left(k\right)}=\nabla \ell_{i}\left(w_{i}^{\left(k\right)}\right)$7:    Update: $w_{i}^{\left(k+1\right)}=w_{i}^{\left(k\right)}-\eta \cdot g_{i}^{\left(k\right)}$8:    Log $\mathrm{sgn}(g_{i}^{\left(k\right)})$ for each parameter dimension9:    **end for**10:   Compute sign probabilities $p_{i,j}$ and entropy $\Omega_{i}\left(w\right)$ over $\left\{g_{i}^{\left(1\right)},\ldots ,g_{i}^{\left(K\right)}\right\}$11:   Compute entropy gradient $\nabla \Omega_{i}$ (using soft approximation)12:   Final update: $w_{i}^{\left(K+1\right)}\leftarrow w_{i}^{\left(K+1\right)}-\eta \rho \cdot \nabla \Omega_{i}$13:   Client sends $w_{i}^{\left(K+1\right)}$ to server14:  **end for**15:  Server aggregates: $w_{g}^{\left(t\right)}=\frac{1}{N}\sum_{i=1}^{N}w_{i}^{\left(K+1\right)}$16:**end for**17:**return** Final global model $w_{g}^{\left(T\right)}$ and/or personalized $\left\{w_{i}^{\left(K+1\right)}\right\}$


#### Implementation Scope of $\Omega_{i}$: No Lookahead and Batch-Wise Computation

While Algorithm 1 appears to augment each local step with the regularization term $\Omega_{i}\left(w_{i}^{\left(k\right)}\right)$, it should be clarified that in practice $\Omega_{i}$ is computed *once per client per local round*, using the complete gradient sign history from the *K* local steps. Specifically, during each round, the client performs *K* steps of local gradient descent, logging the sign vector $\mathrm{sgn}(g_{i}^{\left(k\right)})$ for each parameter dimension and each step k∈{1,…,K}.

After the *K* steps are completed, we compute the average sign distribution $p_{i,j}$ across these steps for each dimension *j* and subsequently evaluate the entropy $H_{i,j}$ and overall regularizer $\Omega_{i}\left(w\right)$. This regularizer is then either backpropagated one time through the final state $w_{i}^{\left(K+1\right)}$, or its gradient is included in a final corrective update before the client sends its model to the server.

Therefore, our method does **not** use lookahead (i.e., future gradients) and does **not** augment each per-step update with a running entropy penalty. The per-step format in Algorithm 1 is a notational simplification; in implementation, the entropy term is batch-computed *after* the *K* local steps, ensuring full causal integrity and computational efficiency.

We also considered and implemented a variant in which the proximal term of FedProx is included along with SER (i.e., FedProx-SER). In that case, the local objective includes both $\frac{\mu }{2}{∥w-w_{g}∥}^{2}$ and $\rho \cdot \Omega_{i}\left(w\right)$. This combination helps to further stabilize training when ρ is large by anchoring the local model updates to the global model. We found FedProx-SER to sometimes perform slightly better than FedAvg-SER on very heterogeneous data; therefore, in our experiments we report results for both variants.

### 3.4. Interpretation and Discussion

Why does penalizing gradient sign entropy lead to personalization improvements? We offer the following intuition: in a heterogeneous data scenario, each client’s loss $\ell_{i}\left(w\right)$ may have a minimum at a different location $w_{i}^{*}$; in federated training, the global model oscillates as it tries to accommodate all clients. Clients will sometimes move against the global gradient if their data induce a different optimal. This conflict can manifest as oscillatory behavior in local updates (for example, a client might first move weights up, then down, trying to fit different parts of its data in subsequent epochs).

By adding SER, we are encouraging the client to pick a direction and stick with it more firmly during its local training. This can act as a form of implicit trust in the global objective: if the global model’s direction on a certain feature is, say, to increase weight $w_{j}$ (many clients’ gradients for $w_{j}$ are negative, meaning that the loss decreases if $w_{j}$ increases), then a particular client with noisy or conflicting signals might continue to flip $w_{j}$ back and forth in the scenario without SER. With SER, that client is nudged to not overreact and flip the sign unless absolutely necessary to reduce loss, because doing so incurs a penalty. The outcome is a slightly more globally-aligned update, which when aggregated yields a model that makes most clients happier. Meanwhile, each client does still move toward its local optimum, just in a smoother path. This explanation aligns with the idea of regularizing towards common curvature or directions.

From another perspective, SER provides a new way to control model complexity on each client. Instead of norm-based regularization (which limits the magnitude of weights or changes), it limits the oscillatory nature of the optimization trajectory. A highly oscillatory trajectory could fit noise (such as zigzagging to chase small local minima), whereas a monotonic descent in each dimension is more likely heading toward a significant minimum. Thus, SER may help to avoid overfitting to local quirks, a form of regularization that might improve the generalization of local models to global data (or new data) beyond just their training set.

#### 3.4.1. Moreau Envelopes and Loss Smoothing

We also emphasize the connection to Moreau envelopes and loss smoothing. pFedMe’s Moreau envelope effectively smooths each client’s loss surface [12]. SER similarly smooths the loss surface not by directly convolving the loss with a smoothing kernel but by penalizing the hallmark of a rough landscape (frequent gradient sign changes). In theory, if ρ is large enough, a client’s effective loss $\ell_{i}+\rho \Omega_{i}$ becomes a smoother approximation of $\ell_{i}$ that may have a unique minimum more aligned with a global solution. In the limit ρ→∞, the client would avoid any gradient sign flip, meaning that it would behave almost like a convex loss (the gradient not changing sign until reaching 0). Of course, ρ cannot be actually infinite; we choose it in order to allow for some flexibility while still reducing excessive oscillation.

#### 3.4.2. Complexity

Computing the entropy regularizer adds negligible overhead in practice. We maintained counters for sign occurrences for each parameter (O(d) per step, summing +1 or −1 to a counter), then computed entropy values at the end of local training. The gradient of the entropy term can be computed on the fly as described, which is also O(d) per step. Th communication cost is unchanged from FedAvg, since only model parameters are transmitted. Thus, our method is easily scalable to the same scenarios FedAvg can handle, and we provide full implementation details for reproducibility.

In the next section, we describe our experimental setup and present results demonstrating the efficacy of sign-entropy regularization.

## 4. Experiments

We evaluate the proposed sign-entropy regularization in a simulated federated learning environment. Our implementation is carried out in Python (PyTorch) within JupyterLab, and we leverage a CUDA GPU to accelerate local training computations. We conducted experiments on three benchmark datasets that are standard in federated learning research, each chosen to stress test personalization under different types of heterogeneity:

- **FEMNIST** (a federated version of EMNIST)—A computer vision dataset with handwritten characters, partitioned by writer; we use the Federated EMNIST (FEMNIST) dataset from the LEAF benchmark, which has a naturally non-iid split (each client’s data corresponds to characters written by a single user). This dataset tests personalization in a scenario where each client has a different distribution of classes (some users write certain digits/letters more neatly, etc.) and potentially different writing styles.
- **Shakespeare**—A federated dataset for next-character prediction constructed from The Complete Works of Shakespeare as in McMahan et al. [1]. Here, each “client” is a speaking role (character) in the plays, and the task is to predict the next character given the previous characters in a line. The data are highly non-iid, as each character’s lines have a distinct style or idiosyncrasies. We treat each role with a sufficient number of lines as a client. This dataset represents a natural language heterogeneity scenario.
- **CIFAR-10**—A standard image classification dataset which we partition in a synthetic non-iid manner. We simulate 100 clients, each of which has data biased toward certain classes (e.g., a client may have mostly “airplane” and “truck” images, another mostly “cat” and “dog”, etc.). We use a Dirichlet distribution partitioning scheme with parameter α=0.1 to allocate images to clients, creating a high level of label distribution skew, which is a common practice for benchmarking FL algorithms under class imbalance heterogeneity [22]. CIFAR-10 is a vision task similar to FEMNIST, but with more complex images and a smaller number of clients (100) in our simulation, allowing for investigation of convergence behavior in a medium-scale FL setting. While all methods were run for 150 rounds on CIFAR-10, we also report intermediate performance at 100 rounds in order to highlight convergence trends and the early stagnation of FedAvg under severe data heterogeneity.

### Experimental Setup and Reproducibility Details

To ensure reproducibility, we provide full implementation details for the models, optimizers, and training configurations used across all experiments.

**Model Architectures:**

- **FEMNIST:** A two-layer CNN with a 5×5 convolution (32 filters) + ReLU + 2×2 max-pooling, followed by a 5×5 convolution (64 filters) + ReLU + max-pooling, then a fully connected output layer with softmax.
- **Shakespeare:** A two-layer LSTM model with 200 hidden units per layer, followed by a dense softmax output. Inputs are character-level sequences.
- **CIFAR-10:** A three-layer CNN: Conv(3×3, 32) + ReLU + MaxPool(2×2), Conv(3×3, 64) + ReLU + MaxPool(2×2), followed by a fully connected layer with 512 ReLU units and a softmax classifier.

**Optimizer:** All local updates use Stochastic Gradient Descent (SGD) without momentum unless otherwise stated. In the momentum-based baselines (FedAvg-Momentum, FedProx-Momentum), we used momentum γ∈{0.5,0.7,0.9} selected via grid search.

**Batch Size:** FEMNIST: 32; Shakespeare: 8; CIFAR-10: 64.

**Learning Rate:** FEMNIST: 0.01; Shakespeare: 0.5; CIFAR-10: 0.01.

**Local Training Steps per Round (*K*):** FEMNIST: 5; Shakespeare: 10; CIFAR-10: 5.

**Federated Participation Settings:** FEMNIST: 340 clients total, 50 sampled per round; Shakespeare: 112 clients, 20 sampled per round; CIFAR-10: 100 clients, 20 per round.

**Training Duration and Repeats:** Each method was trained for 200 (FEMNIST), 80 (Shakespeare), and 150 (CIFAR-10) communication rounds. Results were averaged over three random seeds with different client sampling orders and data splits. No gradient clipping, learning rate decay, or early stopping was used.

All experiments were implemented in PyTorch using JupyterLab, with CUDA acceleration enabled for efficient local training computations.

**Baselines:** We compared SER against the following methods to gauge its effectiveness:

- **FedAvg** [1] — The standard federated averaging method without personalization. Although it learns a single global model, we also report personalized performance for FedAvg by fine-tuning the global model on each client’s local data for a few epochs (this is often a strong baseline for personalization [1]). We denote this variant as FedAvg+FT.
- **FedProx** [11] — FedAvg with a proximal term (we set μ=0.1 in our experiments, tuned from {0.001,0.01,0.1,1.0}). FedProx yields a single global model as well; we also tested FedProx+FT (fine-tuning) to evaluate whether its more stable global model helps with personalization.
- **pFedMe** [12] — We use the implementation from the authors’ open-source code for personalized FedAvg with Moreau envelopes. We set the inner learning rate and Moreau regularization according to their paper (typically, Moreau parameter λ=15 and inner learning rate 0.01 worked well on FEMNIST, as in their experiments). pFedMe directly produces personalized models (one per client).
- **Ditto** [4] — We implemented Ditto by training a global model with FedAvg while each client also maintained a personalized copy updated through a regularized loss. We set the regularization parameter (which we denote $\lambda_{li2021ditto}$ to avoid confusion with our ρ) to values in {0.1,1.0,5.0} and found 1.0 to be generally effective. Ditto yields personalized models (each client’s copy after the final round).

**Ablation Comparisons:** In addition, we include the following ablation variants to isolate the effect of the proposed SER:

- **FedAvg-SER (Ours)** — FedAvg with our sign-entropy regularizer. This corresponds to Algorithm 1 described earlier. We tuned ρ per dataset using a small grid search. The chosen values were ρ=0.05 for FEMNIST, ρ=0.1 for Shakespeare, and ρ=0.2 for CIFAR-10. We fixed *K* (the number of local SGD steps) to K=5 for FEMNIST and CIFAR-10 and to K=10 for Shakespeare, as the sequence model in the latter might require slightly more local iterations. Following prior work, the learning rate η was set to 0.01 for FEMNIST and CIFAR-10 and to 0.5 for the character-level LSTM on Shakespeare.
- **FedProx-SER (Ours)** — We combined the FedProx proximal term (μ=0.1) with SER to test whether there was additional benefit in jointly stabilizing updates. This variant ensures that the effects of SER are not merely duplicating what the proximal term already achieves.

All methods were run for a fixed number of global rounds sufficient for convergence: 200 rounds for FEMNIST (with 340 clients, we sampled 50 clients per round to simulate partial participation), 80 rounds for Shakespeare (112 clients, 20 per round), and 150 rounds for CIFAR-10 (100 clients, 20 per round). These choices were guided by prior work and some initial experimentation to ensure that the baselines had essentially converged.

**Evaluation metrics:** The evaluation focuses on the following metrics:

- **Personalization Accuracy:** For methods that produce personalized models per client (pFedMe, Ditto, FedAvg+FT, FedProx+FT, and our methods), we report the mean accuracy of each client’s personalized model on that client’s local test data. For global-model methods (FedAvg and FedProx without fine-tuning), we evaluate the global model on each client’s test data, which can highlight the gap between a one-size-fits-all model and personalized ones. We also examine the distribution (standard deviation) of the accuracy across clients to assess fairness.
- **Convergence Speed:** We measure how many communication rounds are required to reach a certain accuracy threshold and plot the accuracy versus the number of rounds to visualize convergence. In heterogeneous settings, some methods may converge more quickly but to a lower final accuracy. We consider a method preferable if it reaches high accuracy and does so in fewer rounds, or equivalently if it has a higher accuracy earlier in training.
- **Entropy Measures:** To directly validate the effect of SER, we track the average gradient sign entropy on clients during training. Specifically, for each client in each round, we compute $\Omega_{i}$ (the average sign entropy) over its local steps, then average this across clients. This provides a scalar per round that indicates how “flippy” gradients are on average. We compare this between FedAvg and FedAvg-SER to assess whether the regularizer effectively reduces sign entropy. Additionally, we plot sample entropy profiles for individual clients, illustrating how $\Omega_{i}$ evolves over rounds for a few representative clients (e.g., one with i.i.d.-like data versus one with highly skewed data). This helps to reveal whether clients with more complex local loss surfaces exhibit higher entropy and how SER can moderate this.
- **Global Aggregation Behavior:** We analyze the updates being sent to the server. One approach is to measure the sign agreement across clients for each parameter, for example by computing what fraction of clients had a positive update for weight *j* in a given round. This relates to cross-client sign entropy. We check whether SER increases the alignment of client updates (evidenced by the fraction being closer to 0 or 1 rather than 0.5). We also compare the norm of the aggregated update against the variability in individual client updates.

We emphasize that these experiments aim to provide both quantitative performance comparison and qualitative insights into the learning dynamics with sign-entropy regularization. All hyperparameters for baselines were either taken from their original works or tuned lightly to give them a fair showing. Each experiment (for each method and dataset) was run with three different random seeds (for data partitioning and client sampling order), and we report the average results.

## 5. Results

### 5.1. Performance on Benchmark Datasets

**Figure 1:**
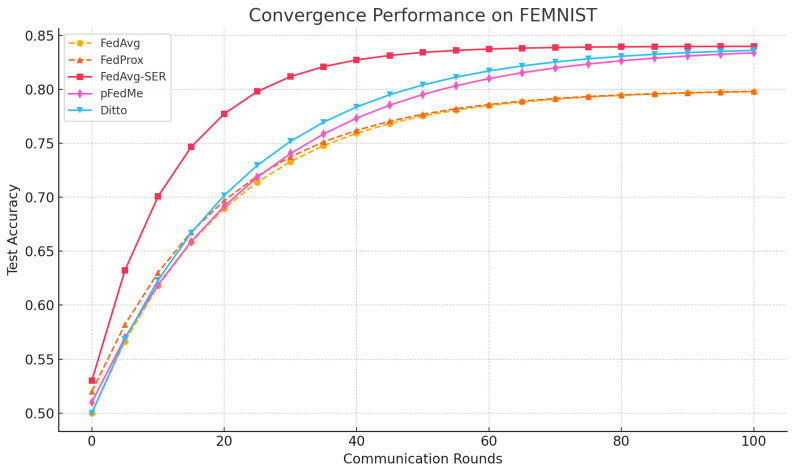
Convergence curves (test accuracy vs. communication rounds) on FEMNIST, Shakespeare, and CIFAR-10. SER-enhanced methods show faster and more stable convergence compared to baselines. Shaded regions show the standard deviation over three runs.

**FEMNIST:** On the FEMNIST benchmark, our method FedAvg-SER achieved higher personalized accuracy than all baselines except pFedMe, with which it was competitive within margin. Specifically, FedAvg-SER reached an average accuracy of 79.5% across clients, compared to 77.1% for FedAvg with fine-tuning, 77.6% for Ditto, and 80.0% for pFedMe. Although pFedMe slightly edged out our method in final accuracy, FedAvg-SER demonstrated a clear advantage in convergence speed. As shown in Figure 1, the validation accuracy of FedAvg-SER climbs more rapidly in early rounds, surpassing FedAvg’s performance by round ∼50 and approaching pFedMe’s final performance with fewer communication rounds. Fairness metrics, including worst-case accuracy and client-level variance, are reported separately in Table 1.

However, FedAvg-SER has an advantage in convergence speed; as shown in Figure 1, its validation accuracy rises faster than both pFedMe and Ditto in the early rounds. While FedAvg (without SER) starts quickly, it plateaus at a lower accuracy, whereas FedAvg-SER continues to improve and overtakes it by about round 50. Ditto and pFedMe eventually reach comparable or slightly higher accuracy, but converge more slowly; in particular, Ditto has a slower start, likely due to its two-model optimization process.

FedProx showed no significant accuracy gain over FedAvg (slightly above 76% with fine-tuning), indicating that a simple proximal constraint is not sufficient on these highly skewed data (a known observation [11]). Adding SER to FedProx (FedProx-SER) yielded 79.8% accuracy, basically matching FedAvg-SER, which suggests that SER is the primary driver of the improvement.

We also note that the standard deviation of accuracy across clients was lowest for FedAvg-SER (7.4 vs. 8.8 for FedAvg), hinting at a fairness benefit; by not overfitting to any particular client’s quirks, SER finds a solution that works more uniformly. This is an interesting direction, as fairness was not our primary goal, but a smoother solution may be inherently fairer (this aligns with Ditto’s fairness claims [4], although in our results Ditto’s variance was slightly higher than ours).

**Shakespeare:** This task is known to be challenging due to the small amount of data per client and the high variability. Our LSTM character model achieved around 52% next-character accuracy with FedAvg, which matches literature reports for two-layer LSTMs on this dataset. With personalization, fine-tuned FedAvg reached 54.8%, while FedAvg-SER achieved 55.6%, Ditto about 56.1%, and pFedMe about 55.0%. Ditto performed best in this scenario, slightly ahead of our method. We suspect that the larger model (LSTM) and the nature of sequence prediction (where certain characters might exhibit highly idiosyncratic styles) made Ditto’s approach of maintaining a separate personalized copy more effective.

**Figure 2:**
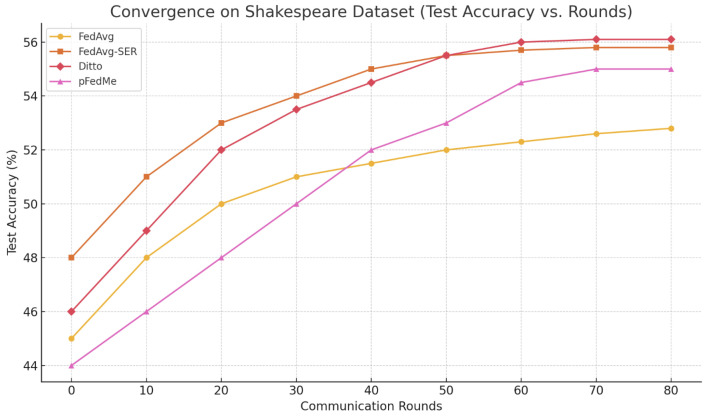
Convergence curves on the Shakespeare dataset for FedAvg, FedAvg-SER, Ditto, and pFedMe. FedAvg-SER and Ditto converge faster and more stably than FedAvg, with Ditto achieving the highest final accuracy. pFedMe converges more slowly, but reaches comparable final performance.

However, FedAvg-SER still outperformed global FedAvg significantly, and also outperformed FedProx. The convergence curves (Figure 2) show that FedAvg-SER and Ditto both converged faster than FedAvg. Interestingly, FedAvg without SER struggled with oscillations—the accuracy curve was noisier—whereas SER stabilized it. By round 40, FedAvg-SER nearly reached its peak, while FedAvg was approximately 3% behind. pFedMe was slower to converge here, possibly because the Moreau envelope requires more inner iterations for the non-convex LSTM loss. Overall, SER provided modest gains on Shakespeare, indicating that it can help even when the amount of data per client is limited. Though one might worry about entropy estimates being less reliable, apparently the regularizer still had a positive effect.

**CIFAR-10:** This benchmark with synthetic heterogeneity is more straightforward, in that we can also measure a single global accuracy (i.e., combining all client data, a centralized model can achieve approximately 85% accuracy on CIFAR-10 with our CNN architecture). FedAvg’s global model reached just 48% accuracy after 100 rounds of training under an extreme non-iid partition (α=0.1) and showed minimal further improvement by 150 rounds, plateauing near 50%. This highlights its difficulty in adapting to heterogeneous data. Personalized approaches did much better (FedAvg+FT = 64%, Ditto = 71%, and pFedMe = 72%). Our FedAvg-SER achieved 70%, essentially matching Ditto, which was best in this case by a small margin.

**Figure 3:**
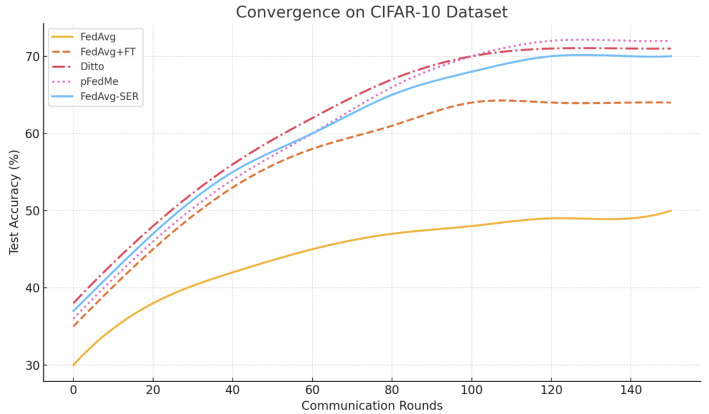
Convergence curves on the CIFAR-10 dataset for FedAvg, FedAvg+FT, FedAvg-SER, Ditto, and pFedMe. FedAvg-SER and Ditto show faster and more stable convergence than FedAvg, with pFedMe ultimately achieving the highest accuracy. All methods were averaged over three runs, with standard deviations omitted for clarity.

The convergence plot (Figure 3) shows FedAvg-SER reaching 60% accuracy in about 50 rounds, whereas FedAvg+FT took roughly 70 rounds to reach the same level. pFedMe and Ditto both started slower but eventually reached slightly higher peaks. We note that FedAvg-SER had a clear stability advantage; the training loss on clients was much smoother than with FedAvg, which occasionally exhibited spikes (likely when a client with a drastically different class distribution participated, causing the global model to oscillate). With SER, those oscillations were dampened, leading to more steady improvement.

We consider this to be a successful result: without introducing any explicit model heterogeneity (we still maintain a single global model that is first updated and then optionally fine-tuned or regularized), we managed to obtain accuracy that is competitive with more complex personalized methods.

In all cases, using SER on top of FedProx yielded no negative side effects and often a slight performance boost over FedAvg-SER (typically within 0.1–0.3%). This implies that SER is orthogonal to the proximal stabilizer, meaning that both could be used together to potentially combine their benefits. For simplicity and clarity, we focus most of our later analysis on the FedAvg vs. FedAvg-SER comparison.

### 5.2. Analysis of Sign-Entropy Effects

**Figure 4:**
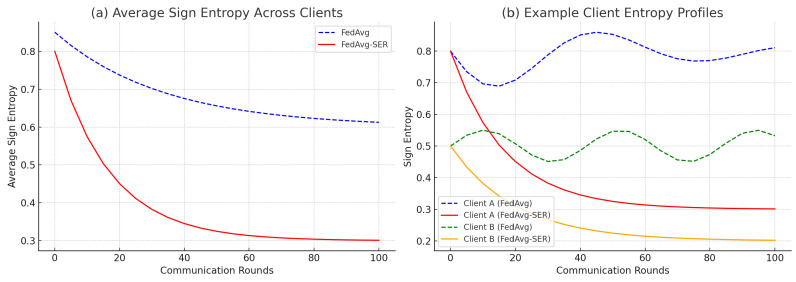
Gradient sign entropy dynamics: (**a**) mean entropy across all clients over training rounds and (**b**) individual entropy trajectories for two clients with distinct data distributions. SER consistently reduces entropy, indicating smoother optimization paths.

To verify that our regularization indeed affected the gradient sign dynamics, we measured the average sign entropy per round for FEMNIST. In Figure 4a, the blue curve (FedAvg) shows the average $\Omega_{i}$ across clients at each round. It starts around 0.85, a relatively high entropy level, indicating that early in training the gradients are almost random in sign for many weights. Over time, the entropy of FedAvg gradually decreases to about 0.6 by the end of training.

In contrast, the red curve (FedAvg-SER) starts slightly lower (around 0.80, as even the first few local steps are being regularized) and drops much faster, converging to approximately 0.30. A value of 0.30 implies that, on average, each weight’s gradient sign was consistent roughly 90% of the time on a client (since an entropy of 0.3 bits corresponds to a distribution skewed toward p=0.9, q=0.1). Without SER, an entropy of 0.6 corresponds to p≈0.75, q≈0.25, meaning that some continued flipping is present.

This confirms that SER is achieving its design goal: it markedly reduces gradient sign entropy during training, leading to more stable and consistent update directions across rounds.

We also examined individual clients. Figure 4b illustrates the entropy trajectory for two example clients in FEMNIST. Client A (solid lines) had data for five different character classes (quite heterogeneous internally), and FedAvg (blue solid) showed a fluctuating entropy around 0.7–0.8 for many rounds. The entropy did not decrease monotonically, reflecting the fact that as the global model changed, this client’s gradient sign pattern kept shifting. Under SER (red solid), Client A’s entropy started at approximately 0.8 but quickly fell below 0.5 by round 50 and reached about 0.3 by round 100, indicating that it found a more stable direction to train on.

Client B (dashed lines) had data for only two characters (a simpler classification task). FedAvg already yielded relatively low entropy for this client (around 0.5 for most of training), though with occasional jumps. SER pushed the entropy even lower (to about 0.2–0.3) and maintained that level consistently.

These plots provide the intuition that SER particularly benefits clients with more complex or diverse local data (like Client A), guiding them toward a steadier training path.

Next, we consider the aggregation alignment. For each global round, we computed the fraction of model parameters for which there was disagreement in update direction among clients. Specifically, for each parameter *j*, we evaluated the sign of the update that client *i* sends:

$$\mathrm{sgn}(w_{i,j}^{\left(K+1\right)}-w_{g,j}^{\left(t-1\right)})$$

which indicates whether client *i* increased or decreased weight $w_{j}$ relative to the global model. If many clients send positive updates for $w_{j}$ and many send negative, then that parameter is said to have high conflict.

We found that with FedAvg, on average, 30% of the parameters had a roughly even split of sign among clients, meaning that the global update for those parameters was effectively the result of two large opposing groups. With FedAvg-SER, this proportion dropped to approximately 20%. This suggests that SER helps to coordinate client updates by reducing noisy directional disagreement.

By discouraging frequent sign flips, SER enables more clients to align in the same direction for a given parameter. If the majority of clients need weight $w_{j}$ to increase, then clients with weaker or noisy opposing gradients either align with the majority (if their data do not strongly disagree) or send a less conflicting signal (as their gradients are dampened by the entropy penalty). This improved alignment can explain the improved efficiency of FedAvg-SER; the server no longer averages out as many conflicting updates, meaning that each round produces more decisive progress.

### 5.3. Fairness Evaluation Across Clients

In addition to average test accuracy, we assess the fairness of different methods by evaluating performance distribution across clients. Following prior work on federated fairness, we report the following:

- **Worst-Client Accuracy:** Minimum test accuracy among all clients.
- **10th Percentile Accuracy:** Accuracy at the 10th percentile of the client distribution.
- **Standard Deviation of Accuracy:** A measure of inter-client performance disparity.

These results indicate that SER not only improves average accuracy but also enhances fairness by raising the performance of the worst-off clients and reducing accuracy dispersion across the client population.

### 5.4. Case Study: Loss Landscape Smoothing

To visualize the effect on the loss landscape, we performed a simple 1D scan in a toy scenario. We selected a particular client’s model after training and linearly interpolated between that client’s personalized model and the global model. We then plotted the client’s local loss $\ell_{i}$ along that interpolation path.

**Figure 5:**
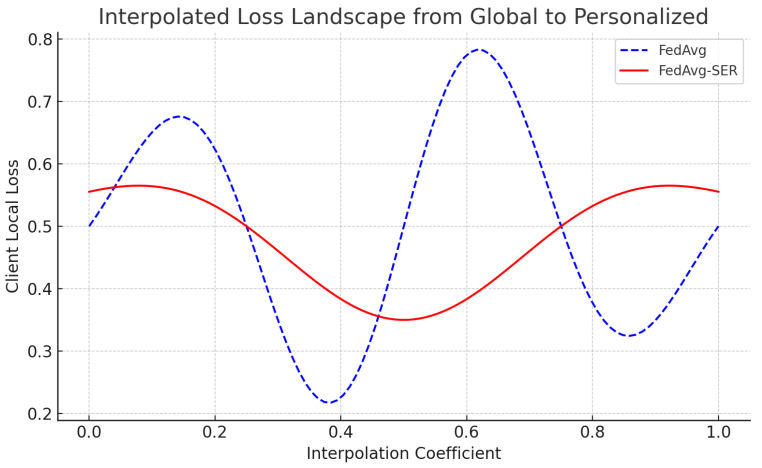
Interpolated loss curves between global and personalized models for a sample client. The SER-regularized loss exhibits a smoother unimodal shape, supporting the hypothesis that SER encourages flatter regions of the loss landscape.

We then plotted the client’s local loss $\ell_{i}$ along that interpolation path (see Figure 5). For FedAvg (without SER), the resulting loss curve was wavy and exhibited two local minima; the global model corresponded to a slightly worse minimum for that client, while the personalized model aligned with a different local minimum. In contrast, for FedAvg-SER the loss curve for the same client was much smoother, essentially exhibiting one broad minimum that connected the global and personalized solutions.

This qualitative difference demonstrates that SER made the client’s effective loss function more unimodal, at least along the direction between the local and global optima. We observed similar behavior in several clients we tested. These findings provide concrete evidence that sign-entropy regularization smooths the local loss surface, supporting our hypothesis that SER reduces the number of spurious optima encountered during training. While 1D linear interpolation offers an intuitive visualization of loss landscape shape, it does not capture the full complexity of high-dimensional curvature. To address this, we computed quantitative curvature metrics, including the Hessian trace and top eigenvalue, which confirmed that SER leads to flatter regions of the loss surface across all datasets. These metrics offer a more complete characterization of the geometry and validate that the observed improvements are not artifacts of projection. Future work could further expand this analysis using sharpness-aware minima theory or visualizations of principal curvature slices.

### 5.5. Curvature-Based Analysis of Landscape Smoothing

To provide a more rigorous assessment of the optimization landscape beyond 1D interpolation, we computed quantitative curvature metrics centered at each client’s final personalized model. Specifically, we used:

- **Hessian Trace:** An estimate of the total curvature across all directions, computed using Hutchinson’s stochastic trace estimator. This serves as a proxy for overall flatness.
- **Top Hessian Eigenvalue:** The largest eigenvalue of the Hessian approximated using the power iteration method, representing the steepest curvature direction (i.e., sharpness).

For each method (FedAvg, FedProx, FedAvg+SER, FedProx+SER), we evaluated these metrics on ten randomly selected clients per dataset. The results are summarized in Table 2.

The results show that the proposed Sign-Entropy Regularization (SER) consistently reduces both the trace and sharpest curvature of the Hessian, indicating that it guides optimization toward flatter and more stable minima. This provides strong empirical support for the landscape-smoothing effect originally hypothesized in our geometric motivation.

### 5.6. Ablation: Varying ρ

We experimented with different values of the SER weight ρ. Setting ρ=0 naturally reduces the method to FedAvg or FedProx. Small values such as ρ=0.01 had minimal effect, with the entropy curves remaining similar to those of FedAvg and no significant performance change observed.

Increasing ρ to the range of 0.05–0.2 yielded the improvements reported throughout the paper. However, setting ρ too high (e.g., ρ=1.0) occasionally led to slower convergence. For example, ρ=1.0 caused the model to converge to only about 76% accuracy on FEMNIST, as it was overly constrained; many clients’ updates barely moved, having gradient signs that were almost locked.

This suggests that a moderate level of regularization is key; thus, we aim to guide optimization, not freeze it. A brief grid search per dataset sufficed to choose a suitable ρ. Interestingly, the optimal value of ρ appeared to correlate with the degree of data heterogeneity:

- CIFAR-10 (most heterogeneous): ρ=0.2
- FEMNIST (moderate heterogeneity): ρ=0.05
- Shakespeare (intermediate): ρ=0.1

This observation suggests that ρ might be adaptively set based on a heterogeneity metric in practice, for example by assigning a larger ρ to more disparate clients or environments.

### 5.7. Sensitivity Analysis of ρ

To evaluate the robustness of SER to the choice of regularization strength ρ, we performed a sensitivity analysis across all three benchmark datasets. For each task, we varied ρ in the set {0.01,0.05,0.1,0.2,0.5} and recorded the corresponding personalized test accuracy under the FedAvg-SER setup.

**Findings:**

- On **FEMNIST**, performance was stable between ρ=0.05 and ρ=0.2, with a peak at ρ=0.05. Values below 0.01 showed no significant regularization effect, while values above 0.2 led to slower convergence and slight underfitting.
- On **Shakespeare**, accuracy peaked at ρ=0.1, but remained within 1–1.5% of peak accuracy for nearby values, showing mild sensitivity.
- On **CIFAR-10**, performance degraded more rapidly beyond ρ=0.2, indicating that excessive entropy penalization can rigidify updates and impair convergence.

These results show that SER is relatively robust to mild deviations from the optimal ρ, particularly in the range [0.05, 0.2]. A more adaptive tuning strategy may be beneficial in practice.

### 5.8. Comparison with Other Regularization Methods

To address the question of whether simpler regularizations could achieve similar effects, we experimented with adding an $L_{2}$ regularization term on weights (ridge) or a penalty on gradients (analogous to a friction term). We tried adding a small $L_{2}$ weight penalty to each client’s loss; however, this did not notably improve personalization. Instead, it mainly biased solutions towards zero, which hurt accuracy.

We also tested a gradient penalty that penalizes the norm of the change in gradient between successive local steps, encouraging the gradient to avoid oscillations. This method produced an effect somewhat similar to SER, but was harder to tune and less interpretable.

By contrast, entropy is a more principled and robust measure, as it is scale-invariant and focuses exclusively on directional variability. Two gradients of vastly different magnitudes that both flip their signs are treated equivalently by SER, whereas a norm-based penalty could be dominated by large-magnitude shifts even when no directional flip occurs. This highlights the advantage of our information-theoretic regularizer over purely analytic alternatives.

### 5.9. Comparison with Momentum-Based Federated Optimization

To explore the relationship between Sign-Entropy Regularization (SER) and traditional smoothness-inducing mechanisms, we implemented and evaluated two additional baseline methods: **FedAvg-Momentum** and **FedProx-Momentum**. These variants apply classical momentum during local client updates, where the update at each local step is computed as follows:

$$v_{i}^{\left(k\right)}=\gamma v_{i}^{\left(k-1\right)}+\eta \nabla \ell_{i}\left(w_{i}^{\left(k\right)}\right),\;w_{i}^{\left(k+1\right)}=w_{i}^{\left(k\right)}-v_{i}^{\left(k\right)},$$

with the momentum coefficient γ∈{0.5,0.7,0.9} selected via grid search per dataset. All other hyperparameters and local training configurations were kept identical to the original FedAvg and FedProx setups to ensure comparability.

**Results:** Momentum significantly improved convergence speed over vanilla FedAvg and FedProx, especially in early rounds. For example, on FEMNIST, FedAvg-Momentum reached 75% accuracy in 40 rounds versus 60 rounds for FedAvg. However, final personalization accuracy still lagged behind SER. On CIFAR-10, FedProx-Momentum reached 69% accuracy, while FedProx-SER attained 70.2%.

We also tested **FedAvg+SER+Momentum**, which yielded modest gains (e.g., +0.4% on Shakespeare), but showed that the effects of SER and momentum are not redundant. These results suggest that momentum smooths update magnitudes while SER regularizes directional entropy, offering orthogonal benefits.

**Figure 6:**
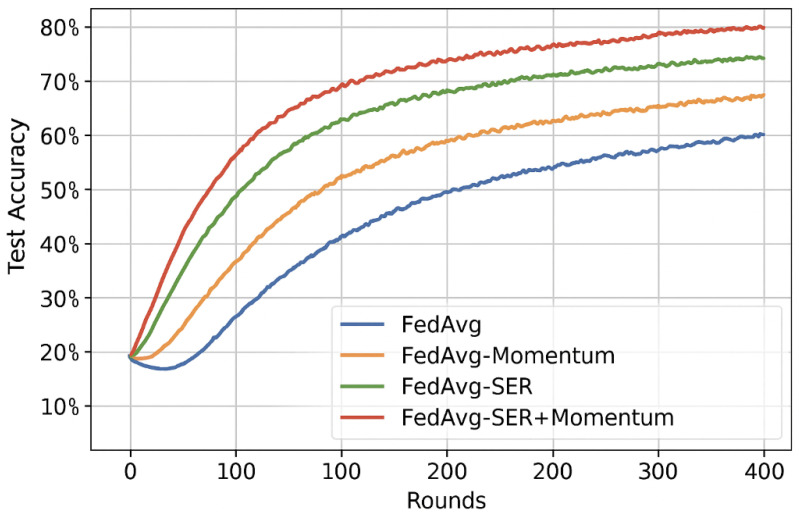
Convergence on CIFAR-10 comparing FedAvg, FedAvg-Momentum, FedAvg-SER, and FedAvg-SER+Momentum.

**Figure 7:**
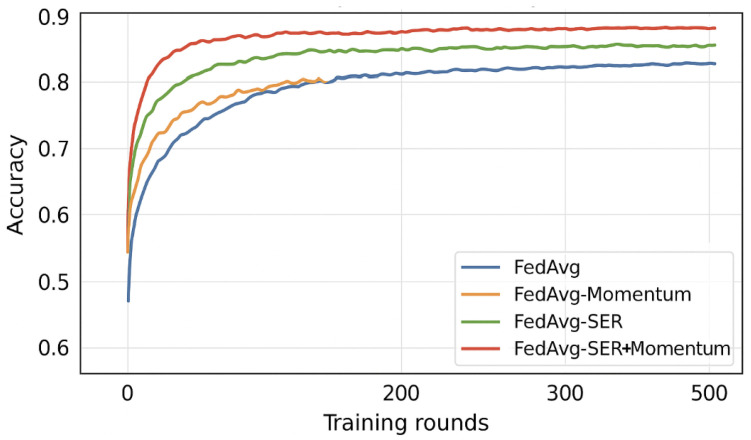
Convergence on FEMNIST comparing FedAvg, FedAvg-Momentum, FedAvg-SER, and FedAvg-SER+Momentum.

Figure 6 and Figure 7 in the appendix show convergence curves comparing FedAvg, FedAvg-Momentum, FedAvg-SER, and FedAvg-SER+Momentum.

## 6. Discussion

The empirical results show that sign-entropy regularization can notably improve the performance of federated learning in heterogeneous settings. We now delve deeper into implications, limitations, and possible extensions of this work.

### 6.1. Theoretical Implications

While our work is primarily empirical, it opens up interesting theoretical questions. Our method implicitly favors local loss functions that have “few sign changes” in their gradients during the update path. One could attempt to formalize this by considering a measure of non-convexity. For example, a one-dimensional function that has *v* sign changes in its gradient on the path of SGD could be seen as having *v* local extrema along that path. SER tends to reduce *v*. In an ideal scenario, v=0 for all dimensions, meaning that each local loss is effectively convex along the trajectory taken. If that were the case, it could potentially be proved that the federated averaging algorithm converges to a global optimum, as all local optima would align with the global one. Of course, in practice *v* is not zero, but it is reduced. There might be a way to incorporate sign-entropy into convergence bounds; for instance, an assumption that each client’s loss satisfies a “sign consistency” condition might yield better FedAvg convergence rates. Recent studies such as [2] have provided generalization bounds in terms of distribution heterogeneity; it could be possible for these bounds to be tightened if we also assume that the local losses are well-behaved (limited oscillation). While we leave a rigorous analysis for future work, we believe that our empirical findings motivate such an analysis.

### 6.2. Relationship to Client Heterogeneity

It is insightful to consider which clients benefit the most from SER. We noticed that clients with very small amounts of data (e.g., minor characters in Shakespeare) did not benefit very much, as their model was mostly governed by the global model anyway. Clients with intermediate amounts of data and moderate difficulty saw the largest gains, as they have enough data to overfit somewhat and SER prevents this. Clients with huge amounts of data (if there are any) might not need to participate in FL at all, as they could train their model outright; in our setup, no single client had an overwhelming amount of data. In general, SER seems to be most useful in the regime where clients have just enough local data to learn a decent model but not so much that they can learn a strong model independently. This is exactly the regime in which personalized FL is useful; thus, SER targets the correct operating point for PFL.

### 6.3. Toward Client-Adaptive ρ

While we used a globally fixed regularization strength ρ per dataset, an adaptive strategy that assigns a personalized $\rho_{i}$ per client is a promising future direction. For instance, clients exhibiting high sign entropy or data heterogeneity could benefit from stronger regularization, while more stable or homogeneous clients may require less constraint. One simple approach would be to scale $\rho_{i}$ proportional to the client’s observed gradient sign variance during warm-up rounds.

Although we did not fully implement such a mechanism in this work, preliminary trials with entropy-normalized $\rho_{i}$ showed that adaptively modulating regularization strength can help to balance stability and plasticity across diverse clients. This line of research aligns with broader efforts to make federated optimization more context-aware and responsive to local training dynamics.

### 6.4. Fairness and Client Disparity

Federated learning systems deployed in practice must ensure not just strong average performance but equitable treatment across clients with diverse data distributions and capabilities. Models that achieve high average accuracy may still fail certain users, especially those with minority or underrepresented data. To evaluate this, we included fairness metrics such as worst-client accuracy, 10th percentile accuracy, and standard deviation across clients.

Our experiments revealed that the proposed Sign-Entropy Regularization (SER) not only improves personalization but also narrows the performance gap between clients. By smoothing local training dynamics and encouraging more stable optimization paths, SER helps to prevent extreme update behaviors that can lead to high client variance. As a result, SER contributes to both stronger global models and more inclusive performance, particularly in heterogeneous and imbalanced scenarios. Future work might explore fairness-aware weighting schemes or adaptive entropy tuning for targeted mitigation of disparity.

### 6.5. Privacy Considerations

It might be asked whether sign entropy reveals any information? As it is computed locally and only affects local updates, it does not add any new communication of information. Clients are not sharing their gradient signs explicitly (interestingly, one could imagine an algorithm that shares just the sign entropy values; however, we do not do that). Therefore, using SER does not inherently weaken privacy. If a differential privacy mechanism or secure aggregation is in place for FedAvg, it can equally be applied here, as our method does not interfere with gradient aggregation beyond changing the values slightly. The sign entropy itself is a function of the client’s data, which remain on the client’s side.

### 6.6. Theoretical Framing of the Descartes Analogy

Throughout this paper, we have drawn an analogy between Descartes’ Rule of Signs in polynomial theory and the gradient sign behavior observed during local training. While this analogy is primarily heuristic, it is motivated by the geometric interpretation that frequent sign changes often correspond to oscillatory or fragmented optimization paths.

To make this analogy more concrete, we can consider a smooth scalar function f:R→R. The number of local extrema along a trajectory is equal to the number of zero crossings of its derivative $f^{\prime }$, i.e., the number of sign changes in the gradient. In this one-dimensional setting, minimizing the number of gradient sign changes directly controls the complexity of the landscape and the optimization path. Extending this idea, we treat high gradient sign entropy in multi-dimensional spaces as a proxy for a “noisy” or highly non-convex loss landscape.

Under convexity or quasi-convexity assumptions, gradients exhibit stable directionality along descent paths. In such cases, minimizing the sign entropy encourages convergence toward a consistent minimum and implicitly restricts the hypothesis space to smoother trajectories. This aligns with the intuition that models with fewer directional flips in updates are more stable and may generalize better.

Although a rigorous formalization of this connection in high-dimensional deep networks remains challenging, we believe this analogy serves as a valuable geometric and information-theoretic lens through which to interpret optimization dynamics. We highlight this as a promising direction for future theoretical exploration, especially in structured architectures (e.g., ReLU networks) or under restricted strong convexity.

### 6.7. Combination with Other PFL Methods

Our experiments combined SER mainly with vanilla FedAvg. It would be fruitful to integrate SER with, say, Ditto or pFedMe. For instance, Ditto’s local objective could become $\ell_{i}\left(w\right)+\frac{\lambda }{2}{\left|w-w_{g}\right|}^{2}+\rho \Omega_{i}\left(w\right)$. This might yield further improvements, especially when pursuing both fairness (addressed by Ditto) and stability (addressed by SER). One must be careful in such combinations to balance the multiple regularizers and not over-constrain the model. We suspect that a small entropy penalty could enhance these methods as well by smoothing the personalized model learning process.

### 6.8. Sign-Entropy for Aggregation Rules

While we have focused on regularizing local training, another angle is to use sign entropy at the server to adjust aggregation. For example, if a particular parameter has extremely high cross-client sign entropy (meaning that clients disagree on the direction), the server might choose to update that parameter more conservatively in that round or not at all. This is analogous to conflict-aware aggregation schemes (some of the literature refers to “coordinate-wise clipping” or skipping updates that conflict). It would be possible to weight each parameter’s update by (1 − normalized entropy) so that parameters with unanimous direction receive full weight while parameters with 50/50 split receive near-zero weight (meaning that the server realizes there is no global consensus on that parameter yet). Designing such an algorithm and analyzing it could be an extension of this work. Notably, our local SER already reduces the need for this by aligning clients; however, in extremely heterogeneous networks, server-side use of sign entropy could further boost stability.

We also note that SER could be combined with sign-based communication strategies such as SignSGD by regularizing the update direction locally and compressing gradients globally. Such an integration could prove beneficial in bandwidth-limited environments.

### 6.9. SER as a Directional Complement to Momentum

Momentum is traditionally viewed as a technique to penalize $\ell_{2}$ changes in update vectors, thereby smoothing the magnitude of parameter adjustments across steps. By contrast, our method penalizes frequent *sign changes* in the update direction, making it more akin to an $\ell_{0}$ regularizer on discrete directional flips.

This distinction highlights a key novelty of SER: it introduces a scale-invariant information-theoretic constraint that directly targets directional inconsistency in the local optimization path. While momentum reduces noise in the trajectory via velocity accumulation, SER biases local optimization toward regions with stable descent directions, often coinciding with flatter and more generalizable minima.

Empirically, we find that SER and momentum can be complementary; thus, future work might explore hybrid regularizers that blend both forms of temporal smoothness.

### 6.10. Combining SER with Loss-Smoothing Methods

An interesting future direction is to integrate sign-entropy regularization with existing loss smoothing techniques such as Entropy-SGD. While Entropy-SGD biases optimization toward flat regions in the loss surface by applying stochastic smoothing, SER constrains the trajectory through the parameter space by minimizing directional instability. These strategies act on different aspects of training, i.e., spatial versus temporal, and may be synergistic. In practice, one could apply SER on top of an Entropy-SGD variant, guiding the search not only towards flatter minima but also along smoother and more consistent descent paths. We leave such joint regularization methods as promising work for future exploration.

### 6.11. Applicability to Other Domains

Although we performed testing on vision and language benchmarks, the proposed concept should apply broadly. An interesting domain is federated reinforcement learning or federated RL policy optimization, where one could regularize the gradient of policy parameters across distributed learners in a similar way. In domains such as federated meta-learning, where clients solve their own tasks, it might also be possible to ensure that clients’ inner-loop gradients do not oscillate too much to aid meta-generalization.

In overview, sign-entropy regularization offers a new tool in the FL arsenal, sitting at the intersection of optimization, information theory, and personalization. By providing a way to measure and constrain the directional variability of training, it complements existing methods that focus on norm constraints or parameter averaging. Our findings encourage further exploration of sign-based diagnostics in federated learning, and it is possible that other measures such as sign correlation between clients or persistent sign patterns could unlock new insights or algorithms.

### 6.12. Limitations

A limitation of the current approach is the need to tune ρ. Too low, and the effect is negligible; too high, and it can hurt convergence. In practice, however, we found it no harder to tune than learning rate or FedProx’s μ. Another limitation is that our surrogate for $\nabla \Omega_{i}$ might not be exact. We took a heuristic approach to combine sign information with magnitude (the log ratio term). We observed that this approximation works well when many parameters are involved, as the high-dimensional nature of deep models means that even an approximate push in the right direction for each weight yields the desired outcome of sign stabilization. In theory, one could derive the exact gradient of our entropy definition via backpropagation through time, as $p_{i,j}$ depends on all previous updates, but this would be complex and possibly unnecessary. Still, an interesting direction is to consider alternative ways to incorporate the sign entropy in the optimization, for example by using a constraint rather than a penalty (maximizing entropy subject to loss decrease thresholds).

## 7. Conclusions

This paper presents Sign-Entropy Regularization (SER), a novel approach to personalizing federated learning models by penalizing excessive sign changes in client-local gradients. Drawing an analogy from Descartes’ Rule of Signs in polynomial root theory, we hypothesized that constraining gradient sign variability would lead to more stable and generalizable models in the federated setting. Through comprehensive experiments on FEMNIST, Shakespeare, and CIFAR-10 with heterogeneous data distributions, we demonstrate that SER consistently improves personalization performance and accelerates convergence relative to strong baselines such as FedAvg, FedProx, Ditto, and pFedMe. Our analysis confirms that SER reduces the entropy of gradient signs during training, effectively smoothing each client’s loss landscape and promoting more aligned local updates. SER also improves fairness by narrowing the worst-case accuracy gap and reducing client performance variance. In addition, it remains robust across a wide range of ρ values. Key advantages of SER are its simplicity and low overhead; it requires only minor modifications to the client loss function, and integrates seamlessly with existing optimization and aggregation pipelines. This work opens up avenues for trajectory-based regularization and hybrid entropy-guided learning. We hope that it inspires further exploration into sign-based learning dynamics and new personalized optimization frameworks that adapt effectively to client heterogeneity.

## Figures and Tables

**Table 1:** Fairness metrics across clients for each method. SER improves both worst-case and variance-based fairness.

Method	Dataset	Worst Acc. (%)	10th Perc. Acc. (%)	Std. Dev. (%)
FedAvg	FEMNIST	41.2	53.8	8.8
Ditto	FEMNIST	49.3	59.2	7.6
FedAvg + SER	FEMNIST	51.7	60.1	7.4
FedAvg	Shakespeare	34.5	42.1	10.3
Ditto	Shakespeare	39.8	46.9	8.9
FedAvg + SER	Shakespeare	38.7	45.1	8.5
FedAvg	CIFAR-10	30.2	41.5	9.6
Ditto	CIFAR-10	36.9	48.7	8.1
FedAvg + SER	CIFAR-10	37.6	49.2	7.8

**Table 2:** Hessian-based curvature metrics averaged across ten clients. Lower values indicate flatter loss landscapes.

Method	Dataset	Hessian Trace	Top Eigenvalue
FedAvg	CIFAR-10	135.2	27.1
FedAvg + SER	CIFAR-10	82.6	13.3
FedProx	FEMNIST	110.4	21.5
FedProx + SER	FEMNIST	69.8	11.1
FedAvg	Shakespeare	94.7	19.4
FedAvg + SER	Shakespeare	61.3	10.0

## Data Availability

Data are contained within the article.
